# Geography and island geomorphology shape fish assemblage structure on isolated coral reef systems

**DOI:** 10.1002/ece3.4136

**Published:** 2018-05-24

**Authors:** Scott Bennett, Andrew R. Halford, J. Howard Choat, Jean‐Paul A. Hobbs, Julia Santana‐Garcon, Anthony M. Ayling, Euan S. Harvey, Stephen J. Newman

**Affiliations:** ^1^ Department of Global Change Research Institut Mediterrani d'Estudis Avançats Universitat de les Illes Balears – Consejo Superior de Investigaciones Científicas Esporles Spain; ^2^ Department of Environment and Agriculture Curtin University Bentley WA Australia; ^3^ Pacific Community Noumea New Caledonia; ^4^ School of Marine and Tropical Biology James Cook University Townsville QLD Australia; ^5^ Sea Research Hydeaway Bay QLD Australia; ^6^ Department of Primary Industries and Regional Development Government of Western Australia Western Australian Fisheries and Marine Research Laboratories North Beach WA Australia

**Keywords:** Christmas Island, Cocos Islands, endemism, island biogeography, multiscale drivers, range edge, reef fish communities, Rowley Shoals

## Abstract

We quantify the relative importance of multi‐scale drivers of reef fish assemblage structure on isolated coral reefs at the intersection of the Indian and Indo‐Pacific biogeographical provinces. Large (>30 cm), functionally‐important and commonly targeted species of fish, were surveyed on the outer reef crest/front at 38 coral reef sites spread across three oceanic coral reef systems (i.e. Christmas Island, Cocos (Keeling) Islands and the Rowley Shoals), in the tropical Indian Ocean (*c*. 1.126 x 106 km^2^). The effects of coral cover, exposure, fishing pressure, lagoon size and geographical context, on observed patterns of fish assemblage structure were modelled using Multivariate Regression Trees. Reef fish assemblages were clearly separated in space with geographical location explaining ~53 % of the observed variation. Lagoon size, within each isolated reef system was an equally effective proxy for explaining fish assemblage structure. Among local‐scale variables, ‘distance from port’, a proxy for the influence of fishing, explained 5.2% of total variation and separated the four most isolated reefs from Cocos (Keeling) Island, from reefs with closer boating access. Other factors were not significant. Major divisions in assemblage structure were driven by sister taxa that displayed little geographical overlap between reef systems and low abundances of several species on Christmas Island corresponding to small lagoon habitats. Exclusion of geographical context from the analysis resulted in local processes explaining 47.3% of the variation, highlighting the importance of controlling for spatial correlation to understand the drivers of fish assemblage structure. Our results suggest reef fish assemblage structure on remote coral reef systems in the tropical eastern Indian Ocean reflects a biogeographical legacy of isolation between Indian and Pacific fish faunas and geomorphological variation within the region, more than local fishing pressure or reef condition. Our findings re‐emphasise the importance that historical processes play in structuring contemporary biotic communities.

## 1 INTRODUCTION

Patterns of species diversity in tropical marine taxa are highly heterogeneous across longitudinal gradients (Tittensor et al., 2010). The most striking examples lie at the boundaries between ocean provinces, in areas which incorporate diversity hotspots and areas where species richness show abrupt changes (Connolly, Bellwood, & Hughes, 2003; Parravicini et al., 2014). These represent situations in which the role of evolutionary and ecological processes in shaping the biotic structure of marine assemblages might be profitably investigated (Cowman, 2014). An important example is to be found at the junction between the Pacific and Indian Oceans partitioned by what is often known as the Indo‐Pacific Barrier (Briggs & Bowen, 2012; Gaither, Toonen, Robertson, Planes, & Bowen, 2010).

**Figure 1:**
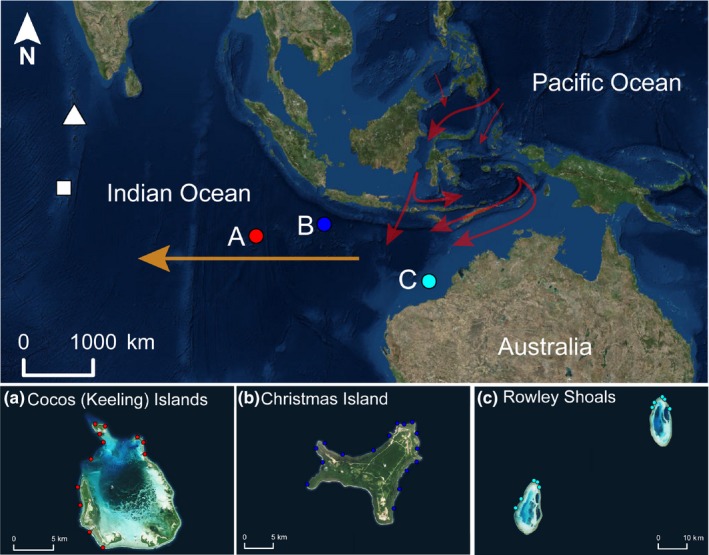
Regional and local maps of study locations at (a) Cocos (Keeling) Islands (red), (b) Christmas Island (dark blue), and (c) Rowley Shoals (light blue), representing the eastern geographical limit for many Indian Ocean fishes and western limit for many Pacific Ocean species. Red and orange arrows indicate the Indonesian flowthrough and the Southern Equatorial Current, respectively. White triangle and square indicate the Maldives and Chagos Archipelago, respectively

The boundary between the western Pacific Ocean and the eastern Indian Ocean is porous due to the influence of varying sea levels on the barrier formed by island chains of the southern Indonesian archipelago (Craig, Eble, Bowen, & Robertson, 2007; Gaither et al., 2010). This barrier is never completely closed, and a major oceanographic feature, the Indonesian throughflow, provides a low‐latitude pathway between the Pacific and the Indian Oceans (Figure 1), transporting warm Pacific Ocean water into the Indian Ocean (Kuhnt, Holbourn, Hall, Zuvela, & Kase, 2004). Biological transport through the Indo‐Pacific barrier is complicated by two factors: (i) the degree of porosity and other environmental conditions of the barrier (e.g., salinity), which have been regulated by fluctuations in sea level, primarily during the Pleistocene; and (ii) taxonomic variation in species ability to move through the barrier, thereby regulating the geographical distribution and genetic structure of coral reef fishes between the two oceans. While some taxa manifest abrupt genetic breaks at this boundary, others have broad geographical distributions extending over both ocean basins with no evidence of breaks in genetic structure throughout this partition (Gaither & Rocha, 2013; Gaither et al., 2010, 2011; Horne, van Herwerden, Choat, & Robertson, 2008; Kennington et al., 2017).

The location of the biogeographical break between Indian and Pacific reef fish populations generally lies to the west of Australia in the vicinity of east longitude 72° (Briggs & Bowen, 2012; Iacchei, Gaither, Bowen, & Toonen, 2016). This biogeographical area, extending from the reef systems of the southern Indonesian island chains and western Australia at approximately 118°E out to the archipelagos of the Central Indian Ocean at 71°E, represents a dynamic region in ecological and evolutionary terms. Over evolutionary time, plate tectonics (Leprieur et al., 2016), and changes in oceanographic processes (especially during the Oligocene–Miocene) have produced habitat configurations that support particularly rich assemblages of shallow water tropical species in the western Pacific Ocean (Keith, Kerswell, & Connolly, 2013; Renema et al., 2008). However, compared with the high abundance of shallow carbonate reef habitats of the western Pacific, the Indian Ocean supports relatively sparse carbonate reef habitats (Spalding & Grenfell, 1997). Connolly et al. (2003) estimate a 43% decline in reef fish species richness over 15° of longitude within this biogeographical area.

Previous studies examining the drivers of reef fish assemblages around the Indo‐Pacific barrier have identified aspects of island biogeography theory, namely, island size and isolation, that help explain patterns of low species richness within remote island reef systems (Hobbs, Jones, Munday, Connolly, & Srinivasan, 2012). However, patterns in species abundance and assemblage structure were poorly explained by island biogeography theory (Hobbs et al., 2012). Long larval duration times and the potential dispersal distances of reef fishes were considered to be prominent reasons for the differences between observed and expected assemblage patterns (Hobbs et al., 2012). In addition, contemporary oceanic processes (Feng, Meyers, Pearce, & Wijffels, 2003; Wainwright, Meyers, Wijffels, & Pigot, 2008) are thought to primarily drive dispersion of larval propagules from east to west (Craig et al., 2007; Hobbs et al., 2009; Hobbs et al., 2010). This may help explain the high proportion and abundances of Pacific Ocean species in the region, despite the closer proximity to the Indian Ocean biogeographical province (Hobbs et al., 2012).

In the context of marine island biogeography, habitat structure, reef history, and geomorphology may also play an important role in shaping fish assemblages on island reef systems. Recent studies have argued that while the capacity for reef species to disperse remains an important feature in the structure of reef assemblages over geographical scales, colonization will only be achieved if propagules find suitable habitats to recruit into (Keith, Woolsey, Madin, Byrne, & Baird, 2015). Therefore, over large temporal scales, establishment may be more important than dispersal for the maintenance of biogeographical breaks in the oceans (Keith et al., 2015). Thus, the influence of geographical location, reef history, and habitat structure could have an important bearing on the composition and functional patterns of faunal assemblages among reef systems.

The purpose of this study was to evaluate the evolutionary and ecological drivers of fish assemblage structure across isolated coral reef systems at the intersection of the Indian and Pacific Ocean biogeographical provinces. These reef systems are important because they represent the western range edge for many Pacific Ocean species and the eastern range edge for many Indian Ocean species as well as overlapping distributions of Pacific and Indian Ocean sister taxa (Choat, klanten, Van Herwerden, Robertson, & Clements, 2012; Hobbs et al., 2014; Sorenson, Santini, Carnevale, & Alfaro, 2013). We focus our study on large (>30 cm) and functionally important reef fishes, as many members of this group are targeted by fishing, thereby enabling the effects of geography, habitat, and human influence on reef fish assemblages to be simultaneously examined. Moreover, large reef fishes, particularly herbivores and piscivorous predators, are arguably the most important groups of reef fish in maintaining the stability of reef systems. The former are an important driver of benthic community composition on coral reefs and often the main mechanistic link between human activity and coral reef decline (Bellwood, Goatley, Brandl, & Bellwood, 2014; Graham, 2015). Meanwhile, larger piscivorous species have been considered significant determinants of the reef fish assemblage structure and activity patterns (Sandin, Walsh, & Jackson, 2010).

This study investigated three reef systems within the eastern Indian Ocean including the Rowley Shoals at 118°E longitude, adjacent to the Indonesian Throughflow, and the oceanic island reef systems of the East Indian Ocean, Cocos (Keeling) Islands (longitude = 96°E), and Christmas Island (longitude = 105°E). The objectives were as follows: (i) to determine the relative importance of multiscale environmental drivers on the structure of reef fish assemblages across the Indo‐Pacific biogeographical boundary. Specifically, we compared the role of geography, reef geomorphology, wave exposure, coral cover, and fishing pressure on the structure of large and functionally important coral reef fishes. (ii) We identify the key species driving taxonomic patterns in reef fish assemblage structure among and within reef systems and (iii) evaluate how taxonomic patterns affect the abundance of key functional groups of fish among reef systems. We discuss the extent to which patterns in reef fish assemblage structure are likely to reflect legacies of contemporary and evolutionary processes, based on phylogenetic and ecological characteristics of species observed across this dynamic biogeographical region.

## 2 METHODS

### 2.1 Geological/geographical context

The study was carried out among three reef systems within an area of the East Indian Ocean bounded by 23° of longitude and 7° of latitude, which encompasses an area of 1.126 × 10^6^ km^2^ of ocean environment. Cocos (Keeling) Islands (CKI), Christmas Island (XI), and the Rowley Shoals (RS) have very different geological histories and geographical locations in the Indian Ocean, albeit with at least 300 km separating each of them from continental land masses (Figure 1). While the three reef systems have perimeters in excess of 50 km, CKI and RS have an atoll configuration. The Rowley Shoals comprise three offshore isolated atoll reefs (Mermaid – 17°06′S, Clerke – 17°19′S and Imperieuse – 17°35′S) on the edge of the continental shelf off western Australia in the easternmost section of the Indian Ocean. Each of the RS reefs has similar dimensions, shape, and orientation and is comprised of a lagoon surrounded by an extensive outer reef flat and crest habitat that rise from the distal ramp of the North West Shelf (Collins, 2011). Similar to the RS, CKI has a lagoon surrounded by a ring of islets and an extensive outer reef flat and crest habitat resting on a limestone plateau. CKI has an age of ~4,000 years since formation of the current reef habitats (Woodroffe & Berry, 1994). In contrast, XI consists of an uplifted limestone cap metamorphosed from coral reefs overlying more ancient volcanic and site bedrock with an age of ~37 mya. Rising from the abyssal Cocos plain, XI lacks a lagoonal system and is surrounded by a narrow shelf and poorly developed fringing carbonate reefs with a vertical profile dropping to depths in excess of 100 m. While the outer perimeter of each reefscape supports healthy reef systems, CKI and RS have approximately 117 km^2^ and 101 km^2^ of shallow reef habitat, compared to XI which has about 34 km^2^ of shallow reef habitat and lacks the extensive lagoonal habitat, which is the major structural feature of CKI and RS (Table 1).

**Table 1:** Marginal tests for predictor groups Spatial and Env. in the DistLM multiple regression

Group	SS(trace)	Pseudo‐*F*	*p*	Prop.	Res. *df*	Regr. *df*
Spatial	1088.8	19.123	.0001	0.52216	35	3
Env.	987.12	7.417	.0001	0.47342	33	5

### 2.2 Fish surveys

To minimize observer bias, all of the fish surveys were conducted by the same observer (A.M. Ayling) between 2004 and 2010. Counts were made at CKI in February 2004, RS (Clerke & Imperiuse Reefs) in October 2007 and XI in March 2010. A total of 38 sites were analyzed consisting of 12 sites at CKI, 16 sites at XI, and 10 sites at the RS (Table S1). For RS, survey data for Clerke and Imperiuse Reefs were combined as they were similar in all measured factors and are within 40 km of each other. All visual census counts were performed as long‐swim belt transects covering an area of (500 × 20 m) and running in a zig‐zag pattern parallel to the reef crest and down to 10 m. All large (>30 TL cm) reef fishes that are often targeted by fishers were counted, with a total species list of 58 species (Table S3).

### 2.3 Environmental factors

Hard coral cover was estimated in a semiquantitative way with four categories representing the range of abundances found on the reefs. Categories were as follows: 0%‐0%, 1‐0 to 15%, 2%‐15% to 30%, 3%‐30% to 50%, and 4%–>50%. Surveys were conducted in XI by A.M. Ayling, CKI by J.P. Hobbs, and the RS survey data were provided by K. Fabricius (Australian Institute of Marine Science). As no quantitative catch data were available for these reef systems, we derived two separate variables to represent estimates of fishing effort. The first used qualitative expert opinion (J.H. Choat, J.P. Hobbs, S.J. Newman) to generate a categorical scale of fishing effort at each site. Categories for perceived fishing effort were as follows: 1 – negligible fishing effort, 2 – low, 3 – medium, 4 – high. The categorical scale of fishing effort was informed by a range of factors including: proximity to populated areas; exposure of the site to prevailing ocean swells; local fisher knowledge from interviews; charter vessel catch data (if applicable); commercial vessel catch data (if applicable); number of fishable days; observations of fishing activity; evidence of fishing activity (e.g., discarded lines etc); and level of compliance activities (if applicable). The second proxy fishing variable was calculated as the distance of each survey site from the nearest port or vessel launching area (DFP) and represents the potential effect of weather on reducing fishing effort at those sites furthest from safe harbor. An exposure index was also calculated for each site by determining the 22.5° sectors (corresponding to traditional compass headings, e.g., E, ENE, NE, NNE, N etc.) from which the site was fully exposed to an unobstructed wave fetch of 3 km or more, and summing the product of the mean annual wind speed at each reef complex (km/hr) and duration (proportion) in each of these sectors (Garcon, Grech, Moloney, & Hamann, 2010). Wind data for all three reef locations were obtained from the Bureau of Meteorology through their web site (http://www.bom.gov.au/climate/data/stations/ : date of access March 2015).

### 2.4 Statistical analysis

Multivariate regression tree (MRT) analysis (De'ath, 2002) was used to explore and model species–environment relationships of reef fish assemblages from CKI, XI, and RS. The fish assemblage matrix was Hellinger transformed prior to running the MRT analysis. This transformation allows for community composition data to be analyzed by metric, Euclidean‐based methods without the problems normally associated with using Euclidean distance as a measure of similarity (Legendre & Gallagher, 2001). In practical terms, it allows for the calculation of an accompanying PCA biplot, which would not be possible if a dissimilarity measure such as Bray–Curtis had been used. MRT's have been shown to be advantageous for dealing with both linear and nonlinear relationships and higher‐order interactions with visual output that allows for ease of interpretation. In addition, explanatory variables do not need to be transformed, and the procedure is robust to collinearity, further aiding interpretation (De'ath, 2002).

**Figure 2:**
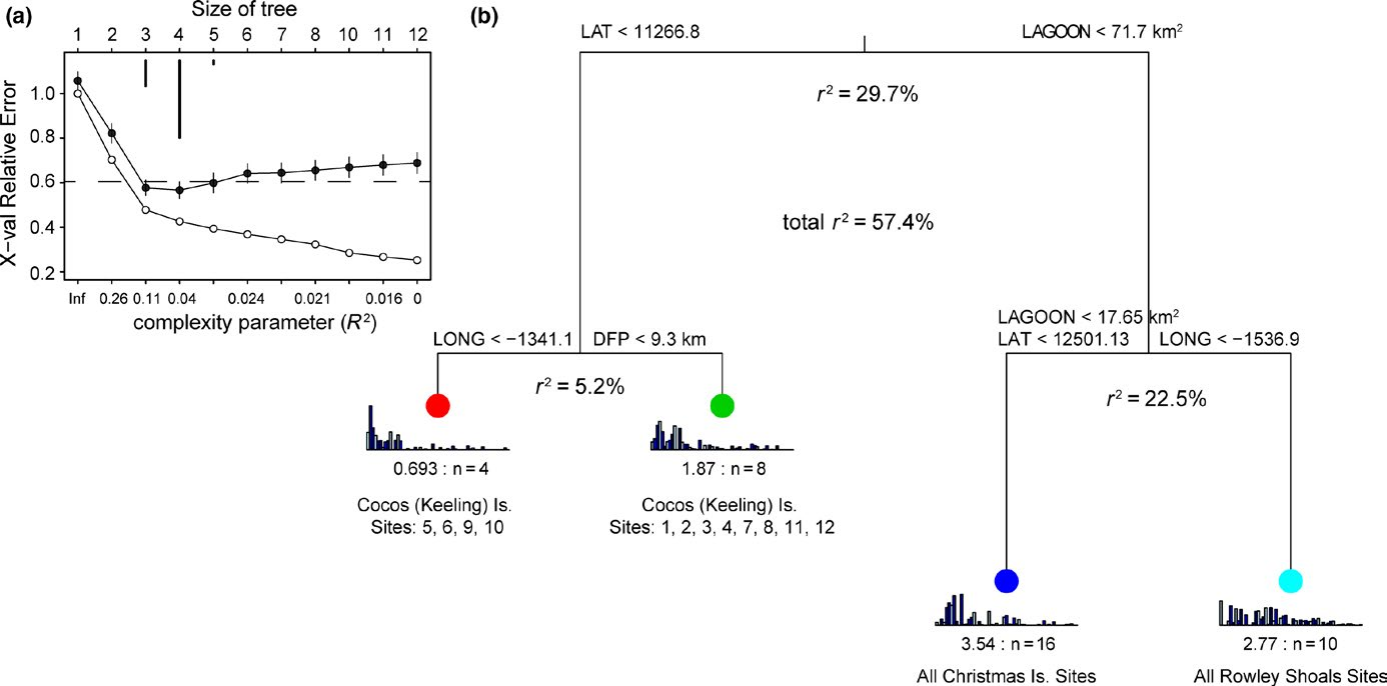
Multivariate regression tree illustrating the role of measured spatial and environmental factors in influencing the structure of fish assemblages across the isolated reef systems of the NE Indian Ocean. (a) Relative error versus complexity parameter indicates a four‐leaf tree was the most parsimonious with 57.4% of variation explained. (b) Multiple explanatory variables were equally plausible at each split indicating that it was not possible to separate such effects from each other. For example, latitudinal differences and lagoon size were equally effective at explaining the observed groupings. Length of the vertical branches is directly proportional to the variance explained. Data were hellinger transformed, and Euclidean distance was used for splitting. Barplots at the bottom of the “leaves” indicate the abundance of species as they occur in the dataset. The colors at the nodes identify the groupings in the PCA biplots that accompany this analysis (Figure 3)

As well as the environmental variables, latitude and longitude were explicitly incorporated into the model to account for spatial autocorrelation, reduce the risk of Type I error, and prevent the spurious inflation of other variables significance—a result we demonstrate using a permutational linear model (Legendre, 1993). The most parsimonious model was selected through 100 cross‐validations of the MRT and selecting of the tree size most frequently calculated as having the lowest standard error (Figure 2a). Indicator values (DLI) were calculated to highlight the species most representative of the groups defined by the MRT analysis (Dufrêne & Legendre, 1997). For a given species at a given grouping, the DLI value takes a maximum of 100 if the species occurs at all sites in the group and nowhere else.

In addition to the MRT analysis, complementary nonparametric linear regression analyses (Linkage Tree and DistLM) were run (Anderson, Gorley, & Clarke, 2008; Clarke, 1993) to compare and assist in validating the results. The Linkage Tree procedure is similar to the MRT process, repeatedly splitting the fish assemblage data into groups according to differences in the explanatory variables. Although not presented here, results were similar between the Linkage Tree analysis and the MRT analysis providing further validation of the MRT model. A distance‐based multiple linear regression (DistLM) was also run using PRIMER software to see how similar the linear model was to the MRT model in terms of explanatory power. Similar *R* ^2^ values indicated that linear relationships were predominant in the model. Explanatory and spatial variables were further pooled into the groups, “env” and “space,” respectively, to demonstrate the importance of controlling for significant spatial correlation when assessing the importance of environmental drivers in multiple regression analyses.

The density of key trophic and taxonomic groups of reef fishes was compared among reef systems (CKI, XI, and RS; fixed factor) using analysis of variance (ANOVA). Species densities were averaged from adjacent sites in CKI and XI, to establish equal sample sizes (*n* = 10 sites) among reef systems. Data were Log+1 transformed to improve normality and homogeneity of variance following visual inspection of scatter plots and the distribution of residuals.

## 3 RESULTS

Regression tree analysis identified that geographical location and/or lagoon size were the biggest predictors of fish assemblage structure, separating the three reef systems, and collectively explaining ~53% of the variation in the dataset and 91% of the model (Figure 2). Sites within CKI were further separated by “distance from port” and/or longitude, which explained 5.2% of the total variation and suggests a possible effect of isolation on fishing between some sites (Figure 2). Other local explanatory variables such as coral cover and wave exposure explained <1% of the variation in assemblage structure and were not included in the most parsimonious tree model. A companion LINKTREE analysis provided similar results helping to validate the MRT results. In addition, a DistLM was run with space controlled for, and this analysis also yielded results very similar to the MRT with a total explained variance of 52.2% versus 57.4%, indicating the predominance of linear relationships. Importantly, environmental variables only explained ~6% of the variation when controlling for spatial structure in the DistLM but this expanded greatly to 47.3% of the variation when spatial structure was removed—highlighting the importance of controlling for the effects of spatial correlation (Table 2).

**Table 2:** Sequential tests for predictor groups Spatial and Env. in the DistLM multiple regression

Group	*R* ^2^	SS(trace)	Pseudo‐*F*	*p*	Prop.	Cumul.	Res. *df*	Regr. *df*
+Spatial	.52216	1088.8	19.123	.0001	0.52216	0.52216	35	3
+Env	.58867	138.69	1.2532	.0689	0.06515	0.58867	31	7

**Figure 3:**
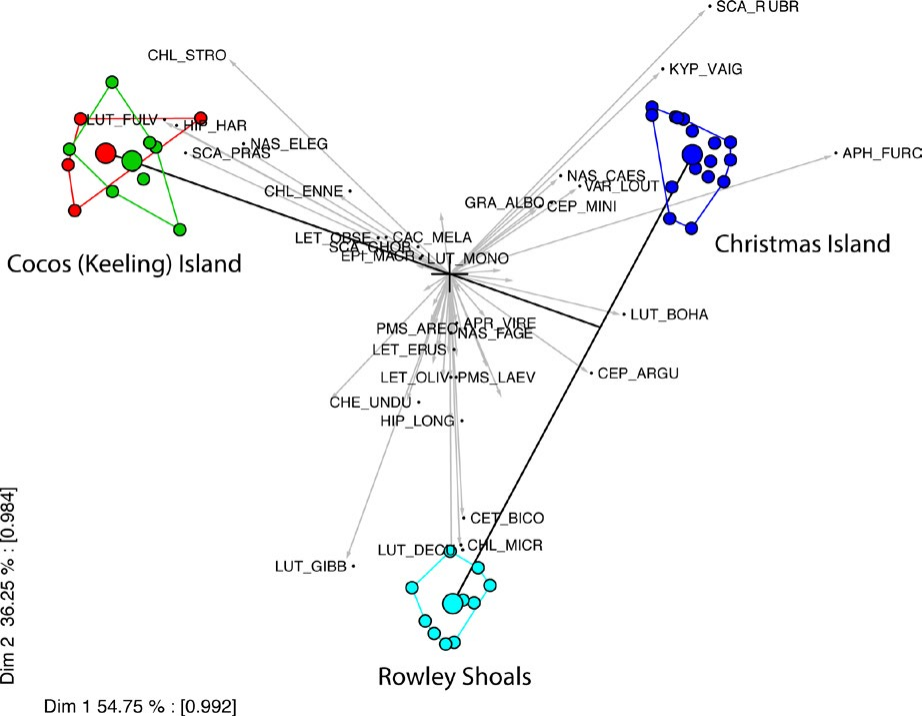
PCA biplot of the first two principal components illustrating the four groups extracted by the MRT analysis. The three reef locations pull out as very distinct from each other and within each reef location sites are clustered close together. The species vectors represent all 45 species underlying the analysis; however, only the 32 found to be significant indicators of the four groups are named

The indicator species analysis identified the species that were significantly associated with each of the groups identified through the MRT analysis (Table 3). The output from the MRT analysis closely resembled the patterns produced by running an unconstrained ordination (such as an nMDS) without explanatory variables. The first split in the tree revealed that the western most atoll group, CKI, was distinguished by high densities of a number of species (e.g., *Hipposcarus harid, Chlorurus strongylocephalus,* and *Naso elegans*) that were replaced by sister species (e.g., *H. longiceps, C. microrhinos,* and *N. lituratus*) to the east (Figure 3, Table 3, Table S2). CKI reefs were also characterized by relatively high densities of excavating and grazing parrotfishes, herbivorous surgeonfishes, and higher‐order predators (e.g., *Carcharhinus melanopterus*) and mesopredators (e.g., *Lutjanus fulvus*) (Table 3, Figure 3). XI assemblages were subsequently distinguished from RS assemblages in the tree by the presence of the small‐medium sized mesopredators such as *Gracila albimarginata*,* Aphareus furca,* and *Variola louti*. In contrast, RS was dominated by different mesopredator species, such as *Lutjanus decussatus* and *Lethrinus olivaceous* (Table 3). The other major distinction between these two systems was the lack of coral trout (*Plectropomus* spp), and the large iconic species *Cheilinus undulatus* and *Bolbometapon muricatum* at XI. The final split identified the species that distinguish between the three main locations and the exposed versus leeward side of CKI. Four species displaying relatively high abundances on isolated CKI reefs were primarily responsible for this division, namely, *Lutjanus fulvus*,* Naso elegans, N. lituratus,* and *Scarus prasiognathus*.

**Table 3:** Table of discriminant species, identified through the IndVal procedure of Dufrene & Legendre (1997)

Regression tree splits	Group Membership	Species	Trophic	IndVal
Cocos‐Keeling (1) v Rest (2)	1	*Hipposcarus harid*	SH	100
Cocos‐Keeling (1) v Rest (2)	1	*Naso elegans*	SH	97
Cocos‐Keeling (1) v Rest (2)	1	*Scarus prasiognathus*	SH	88
Cocos‐Keeling (1) v Rest (2)	1	*Chlorurus strongylocephalus*	EX	86
Cocos‐Keeling (1) v Rest (2)	1	*Lutjanus fulvus*	MP	83
Cocos‐Keeling (1) v Rest (2)	1	*Carcharhinus melanopterus*	P	83
Cocos‐Keeling (1) v Rest (2)	1	*Scarus ghobban*	SH	67
Cocos‐Keeling (1) v Rest (2)	1	*Naso unicornis*	BH	57
Cocos‐Keeling (1) v Rest (2)	1	*Chlorurus enneacanthus*	EX	54
Cocos‐Keeling (1) v Rest (2)	2	*Aphareus furca*	MP	100
Cocos‐Keeling (1) v Rest (2)	2	*Cephalopholis argus*	MP	79
Cocos‐Keeling (1) v Rest (2)	2	*Lutjanus bohar*	P	75
Cocos‐Keeling (1) v Rest (2)	2	*Variola louti*	MP	62
Cocos‐Keeling (1) v Rest (2)	2	*Naso brevirostris*	O	53
Cocos‐Keeling (1) v Rest (2)	2	*Coris gaimard*	I	51
Cocos‐Keeling (1) v Cocos‐Keeling (2)	1	*Lutjanus fulvus*	MP	80
Cocos‐Keeling (1) v Cocos‐Keeling (2)	2	*Naso elegans*	BH	69
Cocos‐Keeling (1) v Cocos‐Keeling (2)	2	*Naso lituratus*	BH	67
Cocos‐Keeling (1) v Cocos‐Keeling (2)	2	*Scarus prasiognathus*	SH	66
Christmas (1) v Rowley Shoals (2)	1	*Scarus rubroviolaceous*	SH	87
Christmas (1) v Rowley Shoals (2)	1	*Kyphosus vagiensis*	BH	79
Christmas (1) v Rowley Shoals (2)	1	*Variola louti*	MP	77
Christmas (1) v Rowley Shoals (2)	1	*Aphareus furca*	MP	75
Christmas (1) v Rowley Shoals (2)	1	*Naso caesius*	O	69
Christmas (1) v Rowley Shoals (2)	1	*Gracila albomarginata*	MP	51
Christmas (1) v Rowley Shoals (2)	1	*Chlorurus strongylocephalus*	EX	50
Christmas (1) v Rowley Shoals (2)	2	*Lutjanus decussatus*	MP	100
Christmas (1) v Rowley Shoals (2)	2	*Chlorurus microrhinos*	EX	96
Christmas (1) v Rowley Shoals (2)	2	*Cheilinus undulatus*	I	96
Christmas (1) v Rowley Shoals (2)	2	*Cetoscarus bicolor*	EX	95
Christmas (1) v Rowley Shoals (2)	2	*Lethrinus olivaceous*	MP	90
Christmas (1) v Rowley Shoals (2)	2	*Lutjanus gibbus*	MP	89
Christmas (1) v Rowley Shoals (2)	2	*Hipposcarus longiceps*	SH	80
Christmas (1) v Rowley Shoals (2)	2	*Plectropomus laevis*	P	80
Christmas (1) v Rowley Shoals (2)	2	*Naso unicornis*	BH	73
Christmas (1) v Rowley Shoals (2)	2	*Coris aygula*	LI	62
Christmas (1) v Rowley Shoals (2)	2	*Lethrinus erythropterus*	MP	60
Christmas (1) v Rowley Shoals (2)	2	*Plectropomus areolatus*	P	60
Christmas (1) v Rowley Shoals (2)	2	*Lethrinus xanthocheilus*	MP	59
Christmas (1) v Rowley Shoals (2)	2	*Bolbometapon muricatum*	EX	50

Only those species with a value greater than 50 are listed. Grouping factor refers to number in parentheses in regression tree splits, identifying where each discriminant species was found Trophic categories: BH, browsing herbivore; SH, scraping herbivore; EX, excavator; I, invertivore; MP, mesopredator; O, omnivore; P, piscivore.

**Figure 4:**
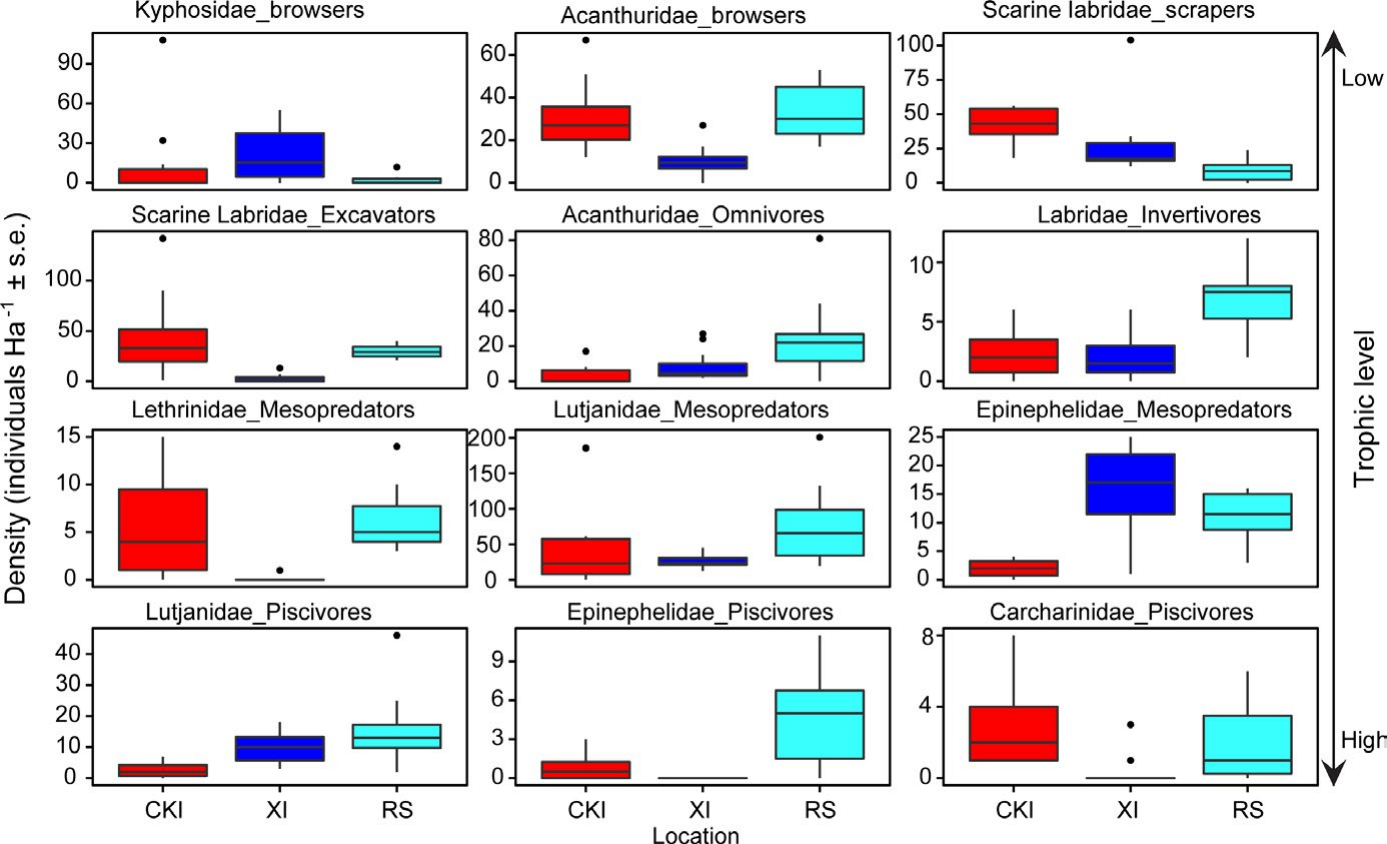
Boxplots illustrating the density of twelve dominant taxonomic and trophic groups among Cocos (Keeling) Island (red), Christmas Island (dark blue), and the Rowley Shoals (light blue)

An overall total of 58 species were recorded in visual surveys, with a relatively even distribution in species numbers between the three reef systems. RS and CKI recorded the equal highest number of species (48) followed by XI (41). However, the assemblage structure of the faunas differed substantially among the reef systems. The differences were driven mainly through absence or rarity of certain taxonomic and trophic groups from different reef systems, particularly at XI (Figure 4). When ordered from east to west across the region, an analysis of variance (ANOVA) revealed that five of the twelve dominant taxonomic and trophic groups display a significant *U*‐shaped pattern in fish density (Figure 4; Table S2). A post hoc Tukey test revealed that browsing herbivores from the family Acanthuridae, excavating Scarine labrids (herein referred to as scarids), lethrinid mesopredators, and carcharhinid piscivores all had significantly lower densities in XI than in both CKI and RS (*p* < .01), while epinephelid piscivores were altogether absent from XI (Figure 4). In the case of browsing acanthurids, lethrinid mesopredators, and epinephelid and carcharhinid piscivores, the U‐shaped density pattern was primarily made up of shared species between CKI and RS (Table S3). For excavating scarids, however, the U‐shaped pattern reflected a high abundance of distinct species, including the sister taxa *Chlorurus strongylocephalus* and *Chlorurus microrhinos* in CKI and RS, respectively (Table S3).

The absence of certain groups from XI was compensated for by higher abundances of other taxa. Browsing herbivores from the family Kyphosidae (20.7 ± 4.9 mean individuals/Ha ± *SE*) and mesopredators from the family Epinephelidae (15.5 ± 1.8 individuals/Ha) displayed higher densities in XI than CKI or RS (Figure 4). Eleven species of epinephelid mesopredators were observed in the study of which *Cephalopholis argus* (4.6 ± 0.8 individuals/Ha), *Variola louti* (4.8 ± 0.82 individuals/Ha)*, Gracila albomarginata* (2.9 ± 0.9 individuals/Ha), and *Cephalopholis miniata* (2.7 ± 0.9 individuals/Ha) were most abundant at XI. Similarly, the large lutjanid piscivore, *Lutjanus bohar*, displayed densities of 9.8 ± 1.2 individuals/Ha, compensating for the rarity of sharks (0.3 ± 0.2 individuals/Ha) and epinepheline groupers (0 ± 0 individuals/Ha) at XI.

The remaining taxonomic and trophic groups displayed a range of density patterns throughout the region. Scraping scarids were the only group to display decreasing density from east to west, driven by high numbers of *Scarus prasiognathus* (19.0 ± 2.7 individuals/Ha) and *Hipposcarus harid* (19.8 ± 2.7 individuals/Ha) at CKI and *Scarus rubroviolaceus* (23.6 ± 5.4 individuals/Ha) at XI. An opposite trend, with numbers increasing from east to west, was seen in the omnivorous acanthurids, resulting from a larger number of shared species (i.e., *Naso brevirostris* and *Acanthurus xanthopterus*) in RS compared with the other regions (Figure 4).

## 4 DISCUSSION

Fish assemblages differed markedly among the coral reef systems across a longitudinal gradient in the eastern Indian Ocean, whereby geography and island lagoon size explained the most variation in fish assemblage structure on reefs (~53%). When examined hierarchically, local‐scale factors such as fishing pressure, coral cover, and wave exposure explained very little variation (~6%) in the fish assemblage structure. Interestingly, if geographical location was not accounted for, then local‐scale factors such as fishing and coral cover explained 47.2% of the variation in assemblage patterns—a result inconsistent with the taxonomic and functional characteristics of the species responsible for differences in the assemblage patterns among reef systems. Major divisions in assemblage structure were driven by sister taxa that displayed little geographical overlap between reef systems and low abundances of several species on Christmas Island that are reliant on the small area of proxy lagoon habitat available within the sole cove of the island. These findings suggest that the geomorphology and geographical legacies of these reef systems are acting as the primary driver of contemporary assemblage structure ahead of fishing pressure or reef condition. Our findings emphasize the importance of adopting a multiscale perspective to understand the underlying mechanisms structuring biotic communities.

The combined influence of geographical isolation and island geomorphology on reef fish assemblage structure around the Indo‐Pacific Barrier builds on previous research that examined fish assemblages through the prism of island biogeography theory (Hobbs et al., 2012). Hobbs et al. (2012) found patterns in species abundance, and fish assemblage structure did not match expectations based on island biogeography theory and cited long larval duration and large dispersal distances by many marine fishes as a possible reason. Our findings support this idea and suggest that in addition to the ability for long‐distance dispersal, the presence of suitable recruitment habitat may also be a fundamental feature for long‐term colonization and the structure reef assemblages across geographical scales (Keith et al., 2015).

The first and major split in the multivariate regression tree separated the most western island (CKI) from the more central (XI) and eastern locations (RS). Geographic location and the effects of lagoon size were responsible for this division and were indistinguishable as proxies for regional‐scale differences in the assemblage structure. Indicator species analysis revealed that the division between CKI and RS/XI was differentiated by a number of abundant sister taxa such as *Hipposcarus harid* vs *H. longiceps*,* Chlorurus stronglyocephalus* versus *C. microrhinos,* and *Naso elegans* versus *N. lituratus*. All three sister taxa have a clear signature of geographical speciation associated with the Indo‐Pacific Barrier (Hobbs et al., 2014), despite having different vicariance events associated with their divergence (Choat et al., 2012; Sorenson et al., 2013). *Naso elegans/N. lituratus* and *C. strongylocephalus/C. microrhinos* diverged during contemporaneous reductions in sea levels associated with Pleistocene glaciation cycles (approximately 2.1 to 2.4 mya, respectively; Choat et al., 2012; Sorenson et al., 2013). The divergence of the *Hipposcarus* species occurred earlier and appears to have been associated with changes in Miocene reef formations (Choat et al., 2012). The division suggests vicariance as the primary evolutionary process shaping assemblage patterns as a consequence of the soft barrier between Indian and Pacific Ocean basins (Choat et al., 2012). Reef fish assemblages in the eastern Indian Ocean are comprised of primarily Indo‐Pacific species with an increasing number of Indian Ocean species occurring toward the west of the ocean basin, many of which have their eastern most range limit around XI or CKI. The geographical context of these islands, both in terms of their isolation and their position at the intersection between the Indian and Pacific Ocean bioregions, therefore has a strong influence on assemblage structure. At a functional level, the sister species driving these patterns share similar feeding modes, thereby reducing the variation in functional composition among islands. Nevertheless, such evolutionary legacies play an important role on the diversity and assemblage structure of contemporary reefs across regional and ocean basin scales.

In parallel to geographical isolation, differences in island geomorphology had an important influence on the fish assemblage structure throughout the region. While all surveys were conducted in comparable coral reef habitats, the size of neighboring lagoon habitats appears to be an important driver of fish assemblage structure on reefs. Lagoon habitats influence both the trophic biology and recruitment patterns of numerous reef fishes (Wilson et al., 2010). CKI and RS have lagoons of 71 km^2^ and 92 km^2^, respectively, with a variety of habitat types ranging from sheltered, algal dominated sites to patch reefs supporting high densities of living coral interspersed with extensive areas of sand and seagrass beds. In contrast, XI comprises a continuous but narrow fringing reef with the only area equivalent to a lagoon habitat being a ~1.2 km^2^ area of sheltered bay (cove) that is dominated by coral, has minimal sand (1%), and lacks seagrass. Some lagoon‐associated fish species occur at low densities in this bay and nowhere else around XI (Hobbs et al., 2010). Consistent with these differences in lagoon size, the XI fish assemblages displayed extremely low abundances of several groups including lethrinids, smaller lutjanids, and wrasses that forage over the sandy substrata targeting benthic invertebrates. In contrast, these represent a signature group at CKI and RS. In addition, XI is notable for the rarity of species that recruit into sheltered lagoonal habitats (Hobbs et al., 2010). These include the large labrids *Cheilinus undulatus* and *Bolbometopon muricatum*, groupers, and reef sharks, especially *Carcharhinus melanopterus* and some epinephelids that are present in much larger numbers at CKI and RS. The continued presence of a number of large rare species at Christmas Island is likely to be dependent on the continued health and protection of this small area of lagoon habitat. These results provide a cautionary note about the influence of local habitat condition on fish assemblage structure. While local coral condition had a relatively small influence on fish assemblage structure in the current study, structural and functional loss of this habitat due to repeated mass coral bleaching across the region (Hughes et al., 2017) could reduce important recruitment grounds, thereby impacting fish assemblages in the future.

The low abundances of several charismatic, fishery targeted, and functionally important species from XI are interesting in the context of this study given the relatively low importance of local‐scale processes in driving the differences in assemblage structure among reef systems. Overfishing is a major driver of depauperate fish assemblages in many locations around the world (Jackson et al., 2001; Jennings & Kaiser, 1998). However, the absence of key species in remote regions is often inferred to result from overfishing, without consideration of the geographical and geomorphological context of the location being examined (Taylor, Lindfield, & Choat, 2015). Indeed, a potential effect of fishing was observed to differentiate isolated and accessible reef areas in CKI. Four species displaying relatively high abundances on isolated CKI reefs were primarily responsible for this division, namely, *Lutjanus fulvus*,* Naso elegans, N. lituratus,* and *Scarus prasiognathus*. In turn, our study also suggests that the scarcity or absence of commonly targeted species, such as *B. muricatum, C. undulatas,* and *C. melanopterus* at XI (Hobbs et al., 2014), may not have been driven by overfishing per se, but rather the lack of suitable nursery habitat. Without considering geographical context and landscape features, such as the lack of lagoon habitat, the absence of species such as *B. muricatum, C. undulatas,* and *C. melanopterus* could be incorrectly interpreted as having been overfished (*c.f*. Ruppert, Travers, Smith, Fortin, & Meekan, 2013).

Reef fish assemblage structure at remote coral reef systems in the tropical eastern Indian Ocean appears to primarily reflect a biogeographical legacy of isolation between Indian and Pacific fish faunas and geomorphological variation among reef systems, rather than local fishing pressure or reef condition. Studies that do not include these evolutionary and large‐scale processes risk attributing too much variation in assemblage structure to local processes and risk overemphasizing the need to manage local factors. Explicit recognition of the relative importance of multiscale processes on assemblage structure, including the evolutionary context of an area, is crucial for the effective management of remote and isolated reef systems.

## CONFLICT OF INTEREST

None Declared.

## AUTHOR CONTRIBUTIONS

SJN, SB, ARH JHC, and ESH conceived the ideas. JHC, AMH, and J‐PAH conducted the fieldwork and collected the data. SB, ARH, and JSG analyzed the data. SB led the writing with assistance from all authors.
