# Supplementary material for: Geography and island geomorphology shape fish assemblage structure on isolated coral reef systems
Source: Ecol Evol. 2018 May 24;8(12):6242–52. doi: 10.1002/ece3.4136 (PMC6024146; doi:10.1002/ece3.4136)
Supplement: Supplementary file 1 [file ECE3-8-6242-s001.docx]

***Supplementary material:* Geography and island geomorphology shape fish assemblage structure on isolated coral reef systems.**

Scott Bennett^1,2^, Andrew R. Halford^3,^ J. Howard Choat^4^, Jean-Paul A. Hobbs^2^, Julia Santana-Garcon^1,2^, Anthony M. Ayling^5^, Euan S. Harvey^2^, Stephen J. Newman^6^

^1^ Department of Global Change Research, Institut Mediterrani d’Estudis Avançats (Universitat de les Illes Balears – Consejo Superior de Investigaciones Científicas), Esporles, Spain

^2^ Department of Environment and Agriculture, Curtin University, Kent Street, Bentley, WA 6102, Australia

^3^ Pacific Community, BP D5 – 98848, Noumea, New Caledonia

^4^ School of Marine and Tropical Biology, James Cook University, Townsville, QLD 4811, Australia

^5^ Sea Research, 20 Rattray Ave, Hydeaway Bay, QLD 4800, Australia

^6^ Western Australian Fisheries and Marine Research Laboratories, Department of Primary Industries and Regional Development, Government of Western Australia, P.O. Box 20, North Beach, WA 6920, Australia

**Table S1:** Coordinates of the survey sites and environmental characteristics used in Regression Tree analysis in Christmas Island, Cocos (Keeling) Islands and Rowley Shoals.

| Reef | Site | Latitude | Longitude | Lagoon size (km2) | Exposure | Hard Coral | Fishing pressure | Distance from port (km) |  |
| --- | --- | --- | --- | --- | --- | --- | --- | --- | --- |
| Cocos-Keeling | C1 | -12.075 | 96.845 | 86.5 | 305.1582 | 1 | 3 | 7.005 |  |
|  | C2 | -12.072 | 96.834 | 86.5 | 111.306 | 1 | 3 | 8.380 |  |
|  | C3 | -12.093 | 96.889 | 86.5 | 1942.829 | 2 | 3 | 5.861 |  |
|  | C4 | -12.083 | 96.839 | 86.5 | 28.42621 | 4 | 3 | 7.304 |  |
|  | C5 | -12.142 | 96.814 | 86.5 | 54.3106 | 3 | 3 | 10.193 |  |
|  | C6 | -12.162 | 96.817 | 86.5 | 49.69061 | 1 | 3 | 12.450 |  |
|  | C7 | -12.111 | 96.829 | 86.5 | 34.42066 | 4 | 4 | 6.965 |  |
|  | C8 | -12.092 | 96.843 | 86.5 | 28.42621 | 4 | 3 | 6.216 |  |
|  | C9 | -12.192 | 96.827 | 86.5 | 240.2427 | 2 | 2 | 16.399 |  |
|  | C10 | -12.209 | 96.843 | 86.5 | 1883.908 | 1 | 1 | 18.977 |  |
|  | C11 | -12.087 | 96.882 | 86.5 | 305.1582 | 2 | 3 | 4.744 |  |
|  | C12 | -12.106 | 96.890 | 86.5 | 1185.846 | 1 | 3 | 7.278 |  |
| Christmas | X1 | -10.445 | 105.561 | 0.2 | 84.97574 | 3 | 3 | 14.086 |  |
|  | X2 | -10.462 | 105.589 | 0.2 | 65.63971 | 4 | 3 | 10.449 |  |
|  | X3 | -10.474 | 105.557 | 0.2 | 149.0779 | 2 | 1 | 19.609 |  |
|  | X4 | -10.508 | 105.533 | 0.2 | 196.1926 | 4 | 3 | 24.246 |  |
|  | X5 | -10.453 | 105.547 | 0.2 | 196.1926 | 3 | 3 | 16.882 |  |
|  | X6 | -10.414 | 105.674 | 0.2 | 166.2816 | 3 | 4 | 1.787 |  |
|  | X7 | -10.416 | 105.685 | 0.2 | 131.4353 | 4 | 4 | 2.977 |  |
|  | X8 | -10.412 | 105.698 | 0.2 | 166.2816 | 3 | 4 | 4.445 |  |
|  | X9 | -10.430 | 105.705 | 0.2 | 1272.7 | 3 | 3 | 6.940 |  |
|  | X10 | -10.453 | 105.706 | 0.2 | 1267.388 | 4 | 3 | 9.803 |  |
|  | X11 | -10.474 | 105.705 | 0.2 | 1443.458 | 4 | 3 | 12.966 |  |
|  | X12 | -10.488 | 105.689 | 0.2 | 1462.794 | 3 | 2 | 15.453 |  |
|  | X13 | -10.516 | 105.678 | 0.2 | 1421.588 | 3 | 2 | 18.880 |  |
|  | X14 | -10.545 | 105.669 | 0.2 | 1421.588 | 4 | 1 | 22.456 |  |
|  | X15 | -10.456 | 105.637 | 0.2 | 104.2507 | 4 | 3 | 5.011 |  |
|  | X16 | -10.433 | 105.662 | 0.2 | 89.94926 | 4 | 4 | 1.475 |  |
| Rowley Shoals | RS1 | -17.254 | 119.361 | 35.1 | 781.16 | 2 | 1 | 3.153 |  |
|  | RS2 | -17.247 | 119.354 | 35.1 | 696.9894 | 2 | 1 | 4.260 |  |
|  | RS3 | -17.283 | 119.377 | 35.1 | 871.9386 | 2 | 1 | 0.529 |  |
|  | RS4 | -17.264 | 119.329 | 35.1 | 940.3207 | 2 | 1 | 8.019 |  |
|  | RS5 | -17.278 | 119.322 | 35.1 | 1077.837 | 2 | 1 | 9.785 |  |
|  | RS6 | -17.497 | 118.951 | 56.9 | 757.856 | 2 | 1 | 5.263 |  |
|  | RS7 | -17.502 | 118.962 | 56.9 | 853.8537 | 2 | 1 | 4.006 |  |
|  | RS8 | -17.517 | 118.968 | 56.9 | 871.9386 | 2 | 1 | 2.126 |  |
|  | RS9 | -17.552 | 118.911 | 56.9 | 940.3207 | 2 | 1 | 12.909 |  |
|  | RS10 | -17.579 | 118.894 | 56.9 | 967.3893 | 2 | 1 | 16.441 |  |

**Table S2:** Results of analysis of variance, comparing densities of key trophic and taxonomic groups among Cocos (Keeling) Islands, Christmas Island and the Rowley Shoals.

| Trophic group | Family |  | Df | Sum Sq | Mean Sq | F value | Pr(>F) |
| --- | --- | --- | --- | --- | --- | --- | --- |
| Browsers | Kyphosidae | Location | 2 | 24.74 | 12.37 | 2.96 | 0.068 |
|  |  | Residuals | 27 | 112.71 | 4.17 |  |  |
|  | Acanthuridae | Location | 2 | 62.94 | 31.47 | 19.58 | <0.001 |
|  |  | Residuals | 27 | 43.4 | 1.6 |  |  |
| Scrapers | Labridae (Scarine) | Location | 2 | 143.75 | 71.88 | 42.8 | <0.001 |
|  |  | Residuals | 27 | 45.34 | 1.68 |  |  |
| Excavators | Labridae (Scarine) | Location | 2 | 135.16 | .58  67 | 31.49 | <0.001 |
|  |  | Residuals | 27 | 57.95 | 2.15 |  |  |
| Omnivores | Acanthuridae | Location | 2 | 78.35 | 39.18 | 6.99 | 0.003 |
|  |  | Residuals | 27 | 151.16 | 5.6 |  |  |
| Invertivores | Labridae | Location | 2 | 23.18 | 11.58 | 15.42 | <0.001 |
|  |  | Residuals | 27 | 20.29 | 0.75 |  |  |
| Mesopredators | Lethrinidae | Location | 2 | 40.22 | 20.1 | 17.56 | <0.001 |
|  |  | Residuals | 27 | 30.93 | 1.14 |  |  |
|  | Lutjanidae | Location | 2 | 125.6 | 62.79 | 14.05 | <0.001 |
|  |  | Residuals | 27 | 120.7 | 4.47 |  |  |
|  | Epinephelidae | Location | 2 | 84.25 | 42.12 | 32.88 | <0.001 |
|  |  | Residuals | 27 | 34.59 | 1.28 |  |  |
| Piscivores | Lutjanidae | Location | 2 | 19.93 | 9.96 | 15.66 | <0.001 |
|  |  | Residuals | 27 | 17.18 | 0.63 |  |  |
|  | Epinephelidae | Location | 2 | 22.73 | 11.36 | 18.67 | <0.001 |
|  |  | Residuals | 27 | 16.44 | 0.6 |  |  |
|  | Carcharinidae | Location | 2 | 9.51 | 4.75 | 7.43 | 0.002 |
|  |  | Residuals | 27 | 17.26 | 0.63 |  |  |

**Table S3:** Reef fish family, trophic group and density (± SE) of species recorded on surveys in Christmas Island, Cocos (Keeling) Islands and Rowley Shoals

| Family | Species | Sp. code | Trophic group | Density (individual Ha_­­_^-1­^) | | |
| --- | --- | --- | --- | --- | --- | --- |
|  |  |  |  | CKI | XI | RS |
| Acanthuridae | *Naso elegans* | NAS_ELEG | Browsers | 12.5 ± 1.94 | 0.18 ± 0.13 | 0 ± 0 |
|  | *Naso lituratus* | NAS_LITU | Browsers | 8.91 ± 1.01 | 8.43 ± 1.15 | 17.8 ± 3.79 |
|  | *Naso tonganus* | NAS_TONG | Browsers | 0 ± 0 | 0.31 ± 0.25 | 0.1 ± 0.1 |
|  | *Naso unicornis* | NAS_UNIC | Browsers | 9.16 ± 3.01 | 0.93 ± 0.34 | 15.4 ± 4.8 |
|  | *Acanthurus xanthopterus* | ACA_XANT | Omnivores | 0.5 ± 0.5 | 0.31 ± 0.17 | 6.5 ± 4.21 |
|  | *Naso brevirostris* | NAS_BREV | Omnivores | 2 ± 1.24 | 2.43 ± 0.68 | 11.9 ± 3.82 |
|  | *Naso caesius* | NAS_CAES | Omnivores | 0.16 ± 0.11 | 4.06 ± 1.72 | 0 ± 0 |
|  | *Naso fageni* | NAS_FAGE | Omnivores | 0 ± 0 | 0 ± 0 | 2.4 ± 1.4 |
|  | *Naso hexacanthus* | NAS_HEXA | Omnivores | 0 ± 0 | 0.87 ± 0.38 | 1 ± 0.78 |
|  | *Naso vlamingii* | NAS_VLAM | Omnivores | 0.83 ± 0.67 | 0.5 ± 0.27 | 3.9 ± 1.48 |
| Carcharinidae | *Carcharhinus amblyrhynchos* | CAC_AMBL | Piscivores | 0.83 ± 0.42 | 0.18 ± 0.13 | 0.9 ± 0.34 |
|  | *Carcharhinus melanopterus* | CAC_MELA | Piscivores | 1.33 ± 0.22 | 0 ± 0 | 0 ± 0 |
|  | *Triaenodon obesus* | TRI_OBES | Piscivores | 0.75 ± 0.42 | 0.12 ± 0.08 | 1.1 ± 0.37 |
| Kyphosidae | *Kyphosus cinerascens* | KYP_CINE | Browsers | 7.08 ± 4.10 | 2.37 ± 1.14 | 1.1 ± 0.67 |
|  | *Kyphosus vaigiensis* | KYP_VAIG | Browsers | 7.25 ± 4.94 | 18.312 ± 4.52 | 1.1 ± 0.64 |
| Labridae | *Cheilinus undulatus* | CHE_UNDU | Invertivores | 1.33 ± 0.43 | 0.06 ± 0.06 | 4.5 ± 0.71 |
|  | *Coris aygula* | COR_AYGU | Invertivores | 0.83 ± 0.32 | 0.25 ± 0.14 | 1.6 ± 0.45 |
|  | *Coris gaimard* | COR_GAIM | Invertivores | 0.16 ± 0.16 | 1.5 ± 0.44 | 1.1 ± 0.34 |
| Labridae (Scarine) | *Bolbometopon muricatum* | BOL_MURI | Excavators | 0.41 ± 0.23 | 0 ± 0 | 4.1 ± 1.65 |
|  | *Cetoscarus bicolor* | CET_BICO | Excavators | 0 ± 0 | 0.12 ± 0.08 | 11.1 ± 1.88 |
|  | *Chlorurus enneacanthus* | CHL_ENNE | Excavators | 15.16 ± 7.96 | 0.37 ± 0.31 | 0 ± 0 |
|  | *Chlorurus microrhinos* | CHL_MICR | Excavators | 0 ± 0 | 0.12 ± 0.12 | 14.3 ± 3.24 |
|  | *Chlorurus strongylocephalus* | CHL_STRO | Excavators | 29.08 ± 5.65 | 1.81 ± 0.64 | 0 ± 0 |
|  | *Hipposcarus harid* | HIP_HARI | Scrapers | 19.83 ± 2.7 | 0 ± 0 | 0 ± 0 |
|  | *Hipposcarus longiceps* | HIP_LONG | Scrapers | 0 ± 0 | 0 ± 0 | 5.6 ± 1.77 |
|  | *Scarus ghobban* | SCA_GHOB | Scrapers | 0.66 ± 0.14 | 0 ± 0 | 0 ± 0 |
|  | *Scarus prasiognathus* | SCA_PRAS | Scrapers | 34.91 ± 16.11 | 0.75 ± 0.38 | 1.2 ± 0.41 |
|  | *Scarus rubroviolaceus* | SCA_RUBR | Scrapers | 3.91 ± 0.64 | 23.62 ± 5.47 | 2 ± 0.77 |
|  | *Scarus xanthopleura* | SCA_XANT | Scrapers | 0 ± 0 | 0.87 ± 0.40 | 0.1 ± 0.1 |
| Lethrinidae | *Lethrinus erythracanthus* | LET_EHUS | Mesopredators | 0 ± 0 | 0 ± 0 | 0.6 ± 0.49 |
|  | *Lethrinus erythropterus* | LET_ERUS | Mesopredators | 0.16 ± 0.11 | 0 ± 0 | 1.9 ± 0.80 |
|  | *Lethrinus harak* | LET_HARA | Mesopredators | 0.33 ± 0.33 | 0 ± 0 | 0 ± 0 |
|  | *Lethrinus obsoletus* | LET_OBSE | Mesopredators | 3.75 ± 1.39 | 0 ± 0 | 0.2 ± 0.13 |
|  | *Lethrinus olivaceus* | LET_OLIV | Mesopredators | 0.08 ± 0.08 | 0 ± 0 | 2.3 ± 0.55 |
|  | *Lethrinus xanthocheilus* | LET_XANT | Mesopredators | 0.83 ± 0.40 | 0.12 ± 0.08 | 1.3 ± 0.39 |
| Lutjanidae | *Aphareus furca* | APH_FURC | Mesopredators | 0 ± 0 | 22.56 ± 2.02 | 4.6 ± 0.65 |
|  | *Lutjanus decussatus* | LUT_DECU | Mesopredators | 0 ± 0 | 0 ± 0 | 14.6 ± 3.25 |
|  | *Lutjanus fulvus* | LUT_FULV | Mesopredators | 68.75 ± 38.21 | 0 ± 0 | 0 ± 0 |
|  | *Lutjanus gibbus* | LUT_GIBB | Mesopredators | 17.66± 8.55 | 1.25 ± 0.99 | 45.5 ± 19.23 |
|  | *Lutjanus kasmira* | LUT_KASM | Mesopredators | 0.16 ± 0.16 | 1.75 ± 1.05 | 10.1 ± 7.03 |
|  | *Lutjanus monostigma* | LUT_MONO | Mesopredators | 1.33± 0.55 | 0.06 ± 0.06 | 0.1 ± 0.1 |
|  | *Lutjanus rivulatus* | LUT_RIVU | Mesopredators | 0 ± 0 | 0.12 ± 0.08 | 2.5 ± 1.43 |
|  | *Symphorus nematophorus* | SYM_NEMA | Mesopredators | 0 ± 0 | 0 ± 0 | 0.2 ± 0.2 |
|  | *Aprion virescens* | APR_VIRE | Piscivores | 0.08 ± 0.08 | 0.18 ± 0.13 | 1.1 ± 0.37 |
|  | *Lutjanus bohar* | LUT_BOHA | Piscivores | 2.58 ± 0.74 | 9.81 ± 1.26 | 14.8 ± 3.93 |
| Epinephelidae | *Aethaloperca rogaa* | AET_ROGA | Mesopredators | 0 ± 0 | 0.12 ± 0.08 | 0.3 ± 0.15 |
|  | *Cephalopholis argus* | CEP_ARGU | Mesopredators | 0.33 ± 0.18 | 4.56 ± 0.82 | 9.5 ± 1.26 |
|  | *Cephalopholis miniata* | CEP_MINI | Mesopredators | 0 ± 0 | 2.75 ± 0.91 | 0 ± 0 |
|  | *Epinephelus macrospilos* | EPI_MACR | Mesopredators | 0.58 ± 0.22 | 0 ± 0 | 0 ± 0 |
|  | *Epinephelus spiloticeps* | EPI_SPIL | Mesopredators | 0 ± 0 | 0 ± 0 | 0 ± 0 |
|  | *Gracilia albomarginata* | GRA_ALBO | Mesopredators | 0.16 ± 0.11 | 2.93 ± 0.92 | 0.2 ± 0.2 |
|  | *Variola albimarginata* | VAR_ALBI | Mesopredators | 0 ± 0 | 0.25 ± 0.14 | 0 ± 0 |
|  | *Variola louti* | VAR_LOUT | Mesopredators | 0.91 ± 0.28 | 4.87 ± 0.82 | 1.3 ± 0.44 |
|  | *Epinephelus fuscoguttatus* | EPI_FUSC | Piscivores | 0.75 ± 0.30 | 0 ± 0 | 0.1 ± 0.1 |
|  | *Epinephelus polyphekadion* | EPI_POLY | Piscivores | 0 ± 0 | 0 ± 0 | 0.5 ± 0.5 |
|  | *Epinephelus tauvina* | EPI_TAUV | Piscivores | 0.08 ± 0.08 | 0 ± 0 | 0.2 ± 0.2 |
|  | *Plectropomus areolatus* | PMS_AREO | Piscivores | 0 ± 0 | 0 ± 0 | 1.2 ± 0.57 |
|  | *Plectropomus laevis* | PMS_LAEV | Piscivores | 0 ± 0 | 0 ± 0 | 2.8 ± 0.78 |
